# Supplementary material for: PM10 and Other Climatic Variables Are Important Predictors of Seasonal Variability of Coccidioidomycosis in Arizona
Source: Microbiol Spectr. 2022 Mar 23;10(2):e01483-21. doi: 10.1128/spectrum.01483-21 (PMC9045372; doi:10.1128/spectrum.01483-21)
Supplement: SUPPLEMENTAL FILE 1 — Supplemental material. Download SPECTRUM01483-21_Supp_1_seq7.pdf, PDF file, 1.8 MB [file spectrum01483-21_supp_1_seq7.pdf]

Supplemental Table 1:

Summary of covariates within each test model

Model	*Covariates*	*Spline*	*Smoothing Parameter*
No Seasonality	Wind Speed, Precipitation, Maximum Temperature, PM10	NA	NA
Precipitation	Precipitation	Cyclic Cubic Regression	5
Precipitation/Temperature	Precipitation, Maximum Temperature	Cyclic Cubic Regression	5
All Variables	Wind Speed, Precipitation, Maximum Temperature, PM10	Cyclic Cubic Regression	5
Lagged	Wind Speed, Precipitation, Maximum Temperature, PM10,Lagged Precipitation, Lagged PM10	Cyclic Cubic Regression	5

Supplemental figures:

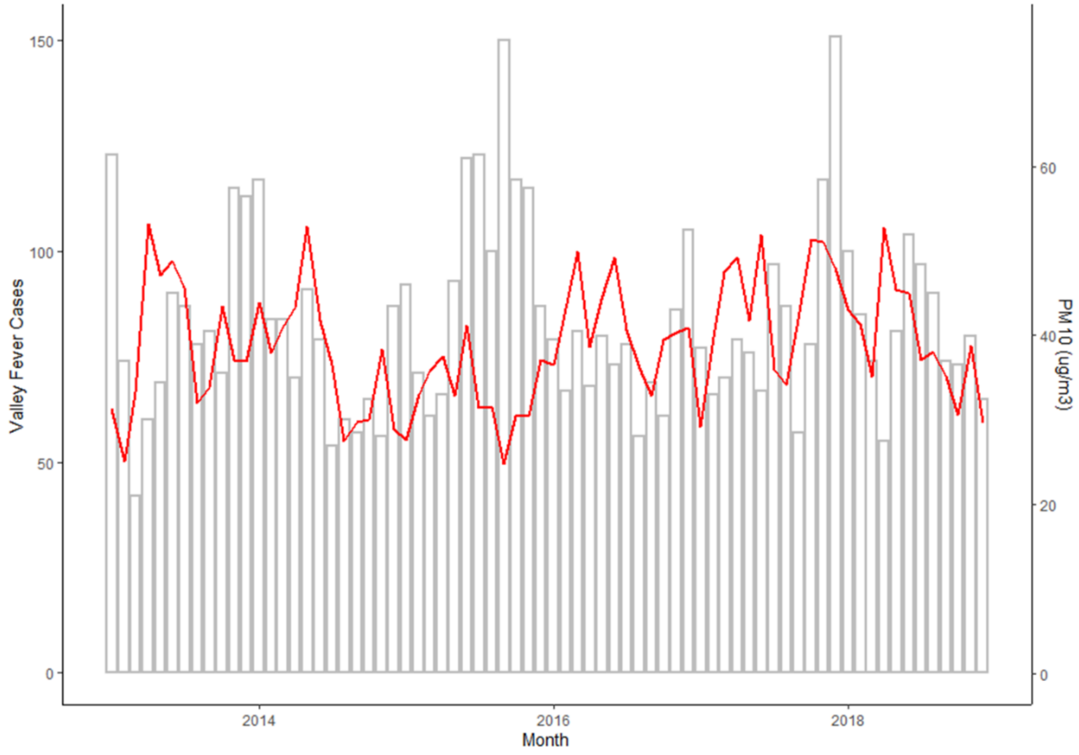

S1a.

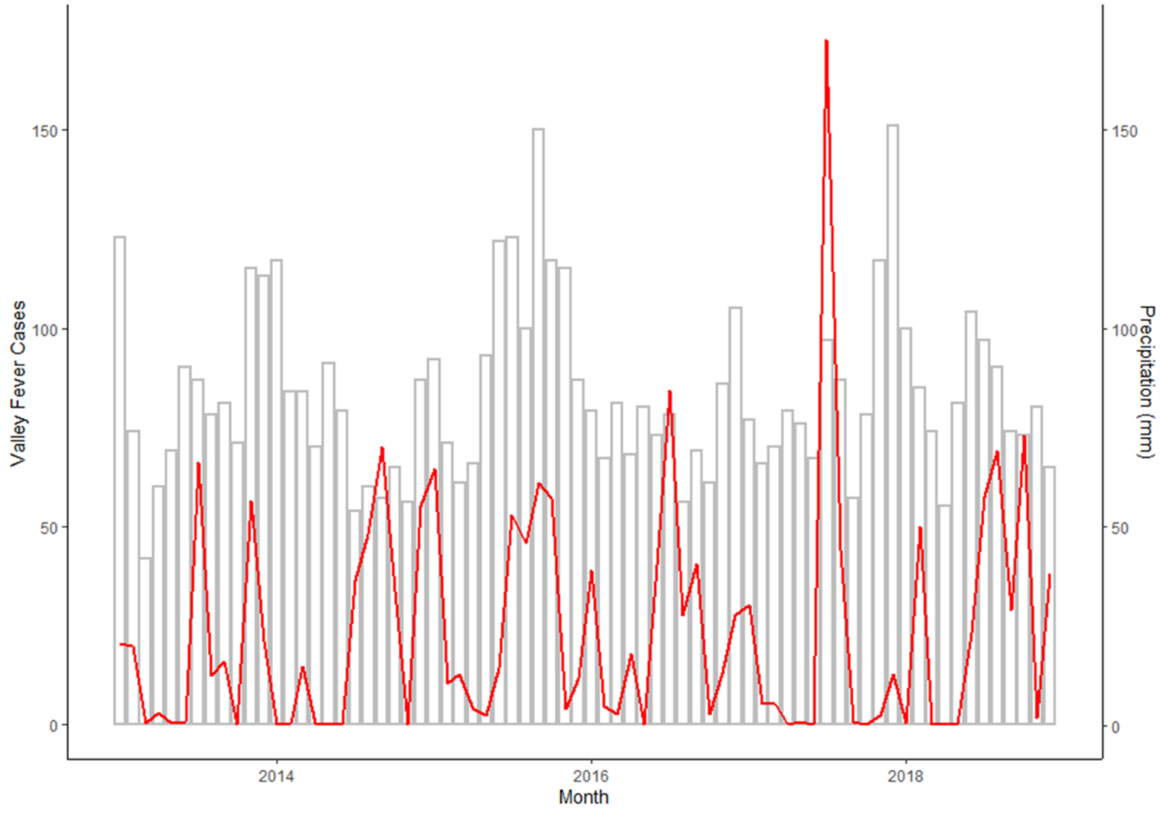
Valley Fever Cases
Precipitation (mm)
Month
2014
2016
2018
0
50
100
150

S1b.

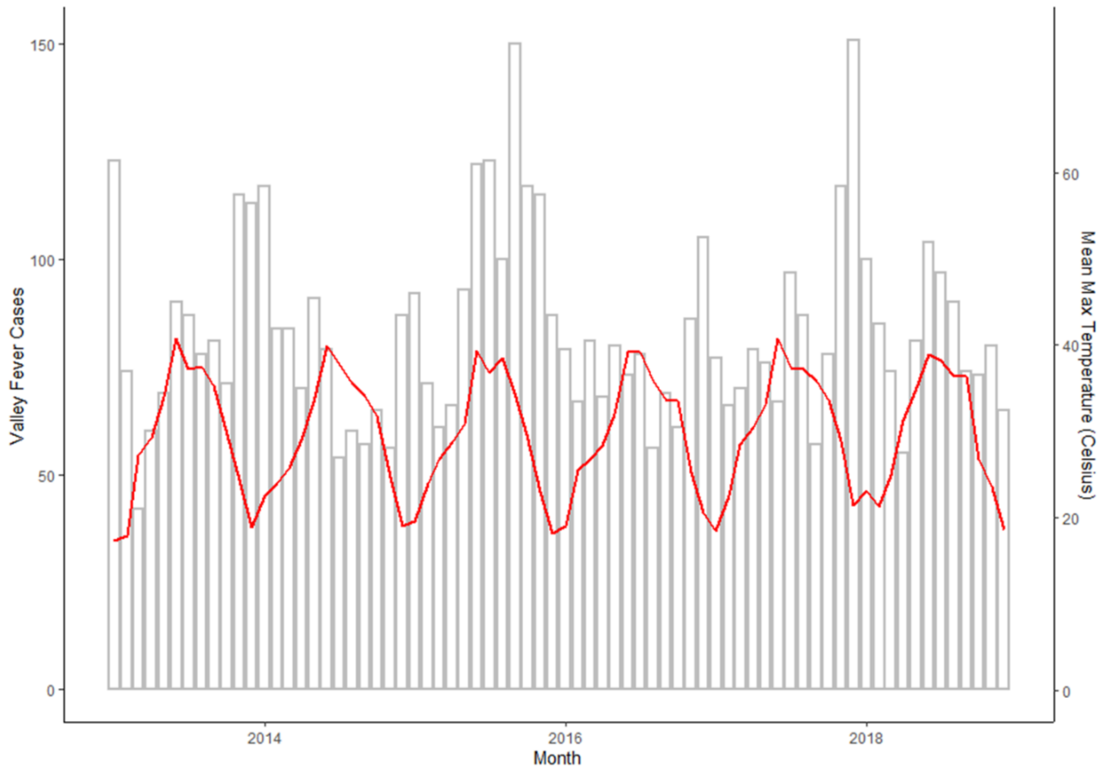
Valley Fever Cases
Mean Max Temperature (Celsius)
Month
2014
2016
2018
0
50
100
150
20
40
60

S1c.

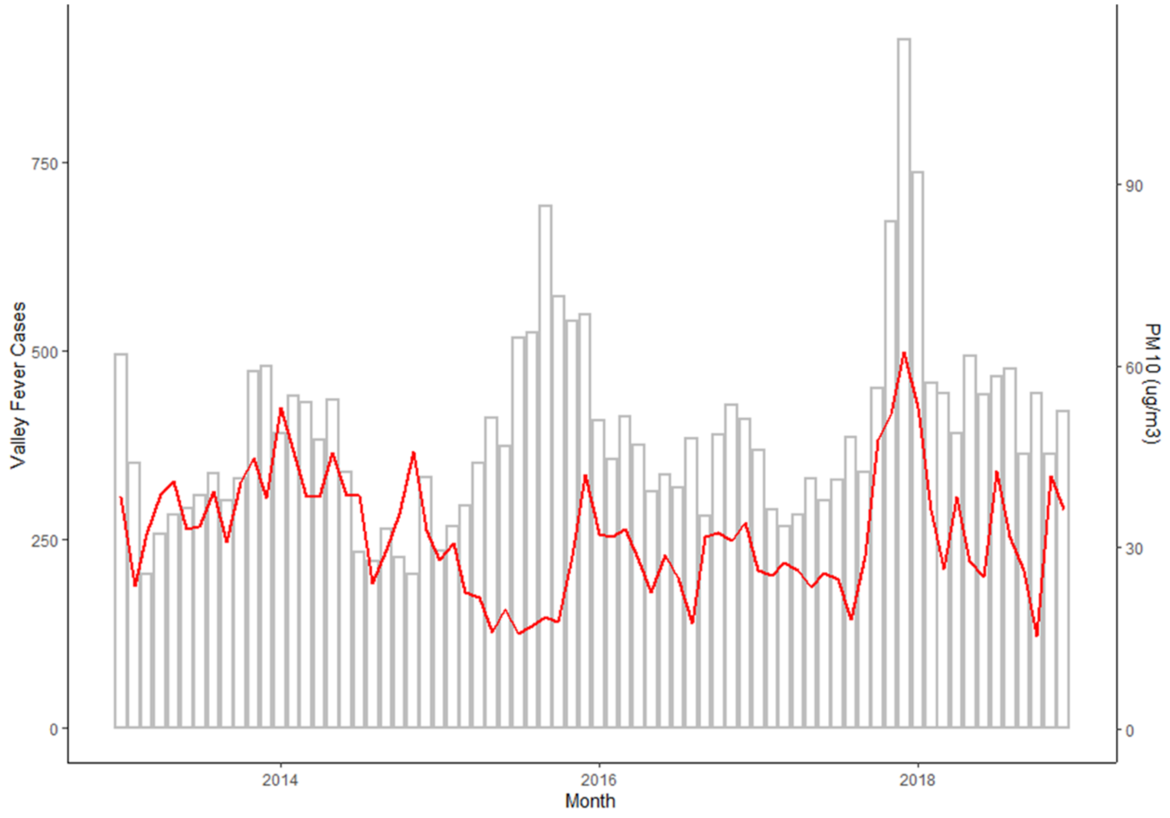
Valley Fever Cases
750
500
250
0
PM10 (ug/m3)
90
60
30
0
2014
2016
2018
Month

S1d.

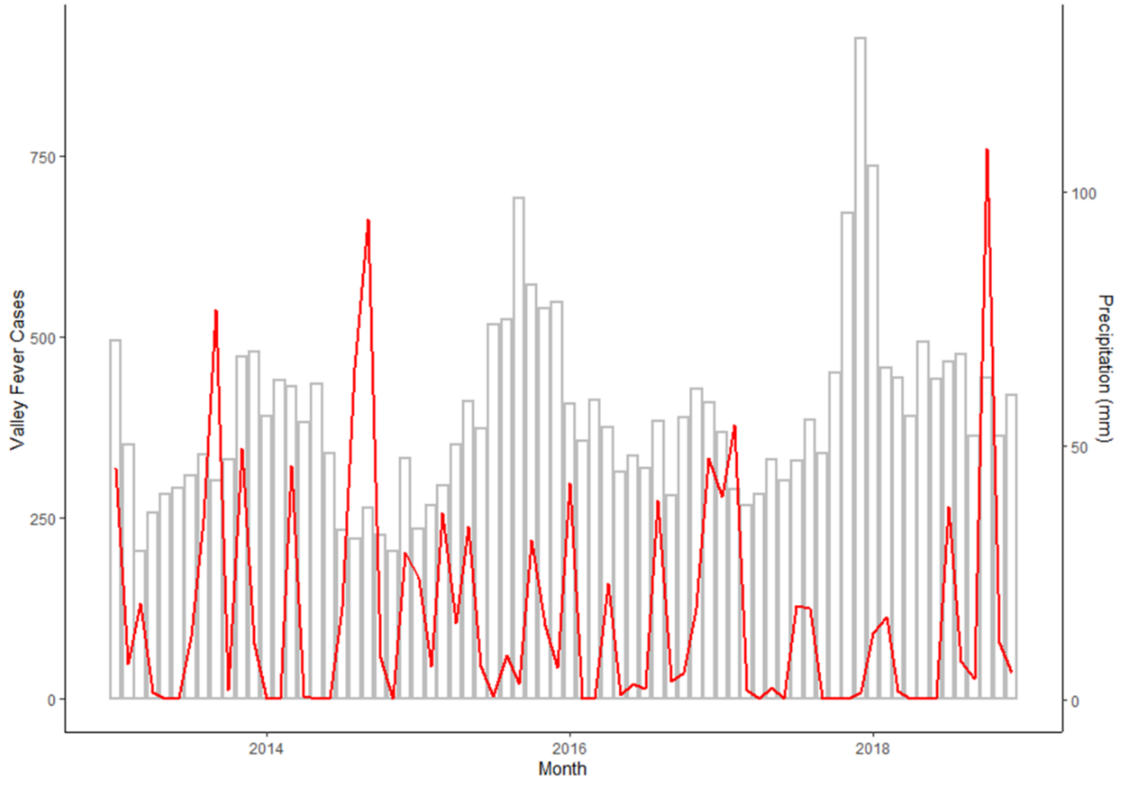
Valley Fever Cases
750
500
250
0
Precipitation (mm)
100
50
0
2014
2016
2018
Month

S1e.

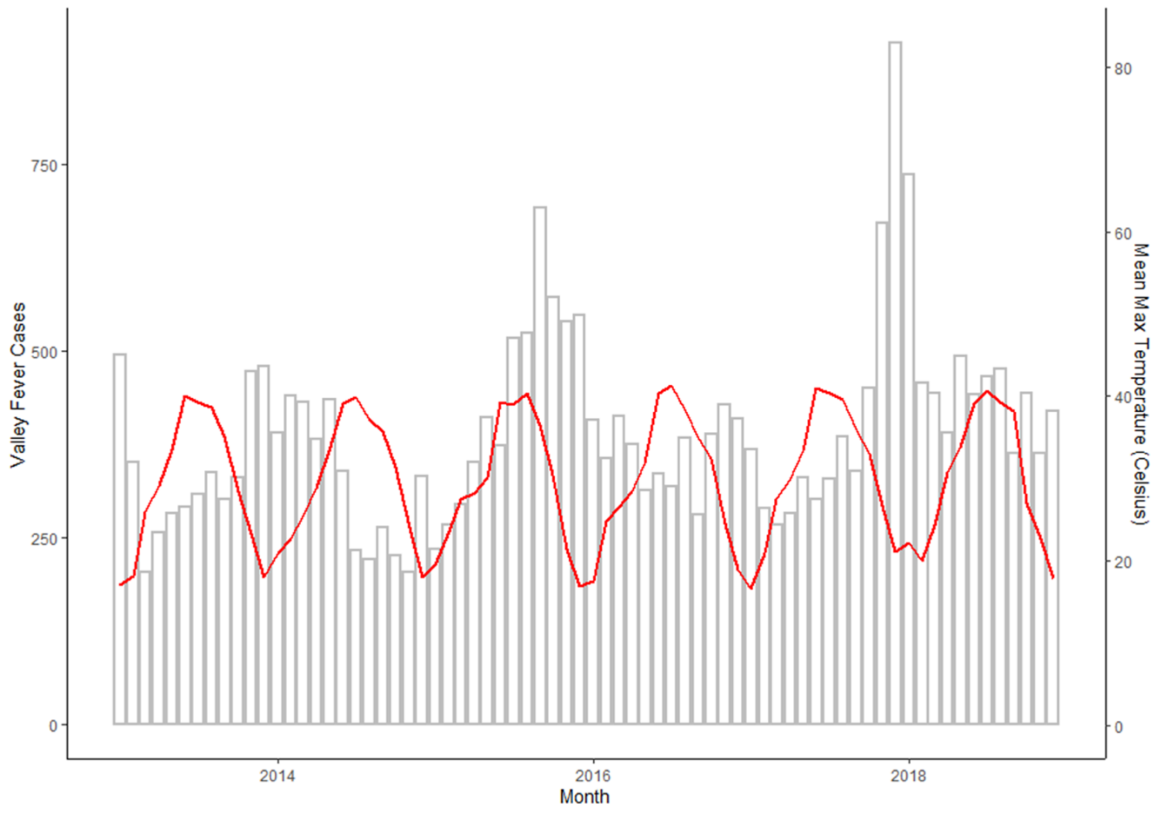

Valley Fever Cases
Mean Max Temperature (Celsius)
Month
0
250
500
750
20
40
60
80
2014
2016
2018

S1f.

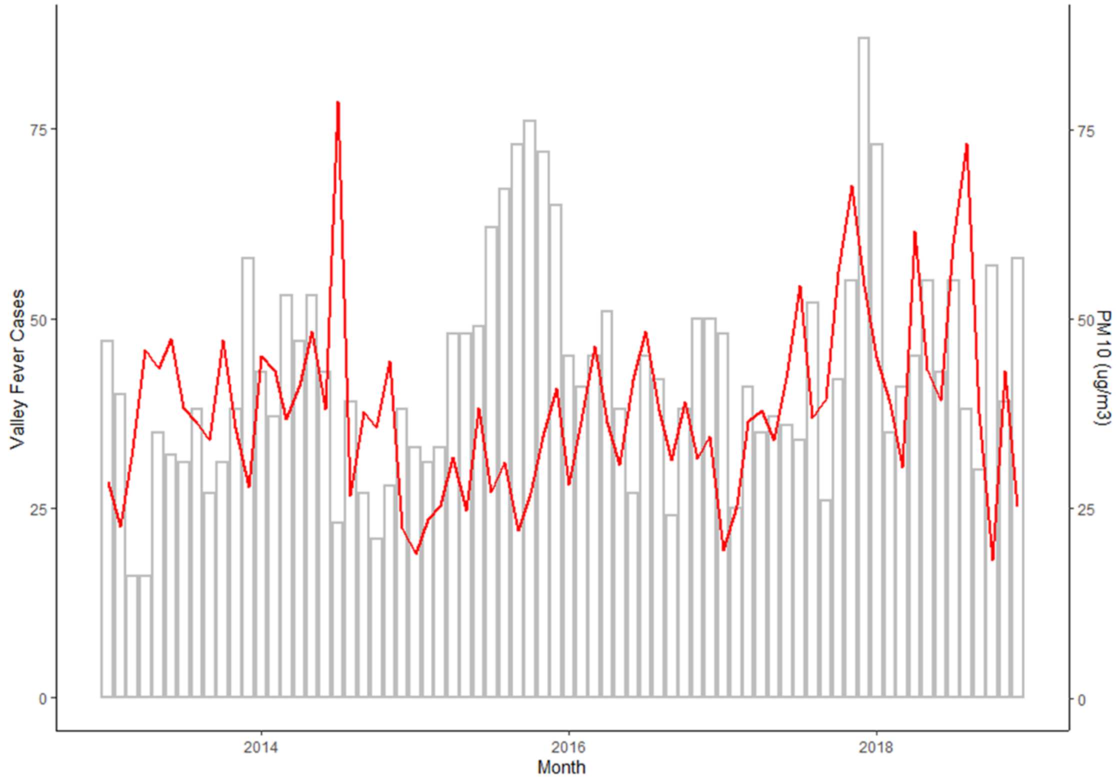

Valley Fever Cases
PM10 (ug/m3)
Month
0
25
50
75
2014
2016
2018

S1g.

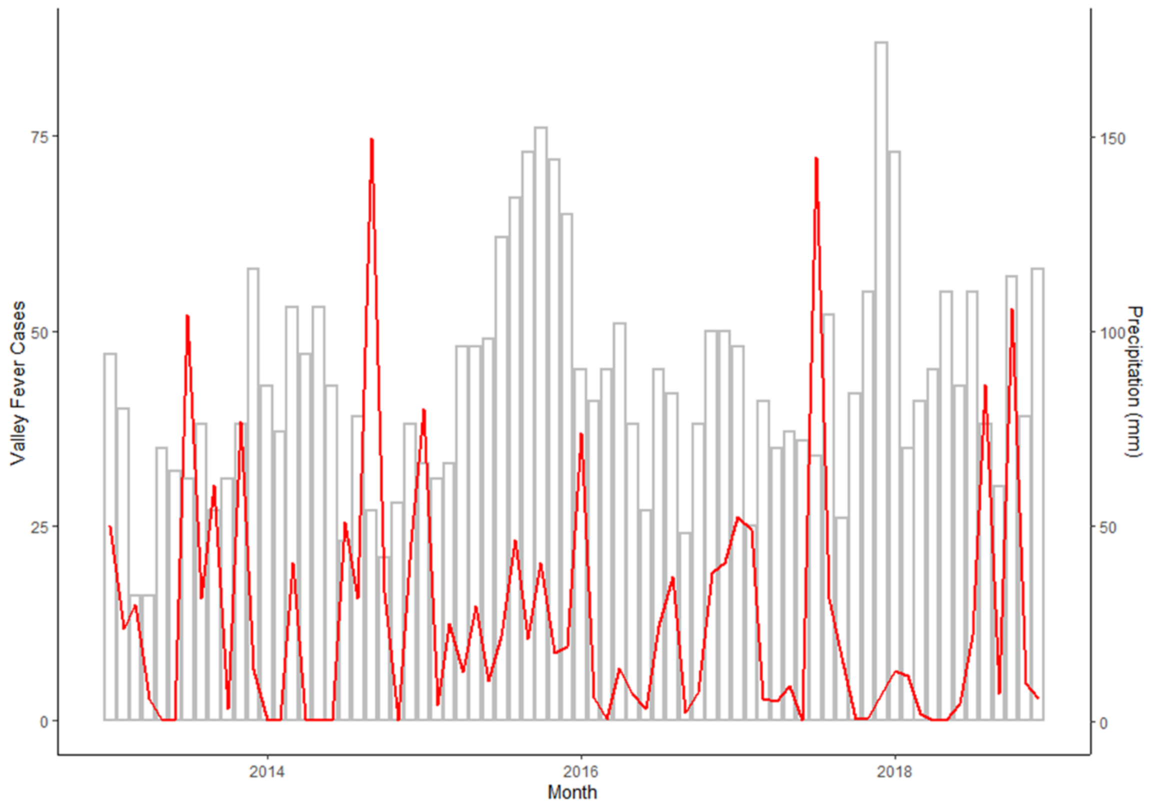
Valley Fever Cases
0
25
50
75
Precipitation (mm)
0
50
100
150
2014
2016
2018
Month

S1h.

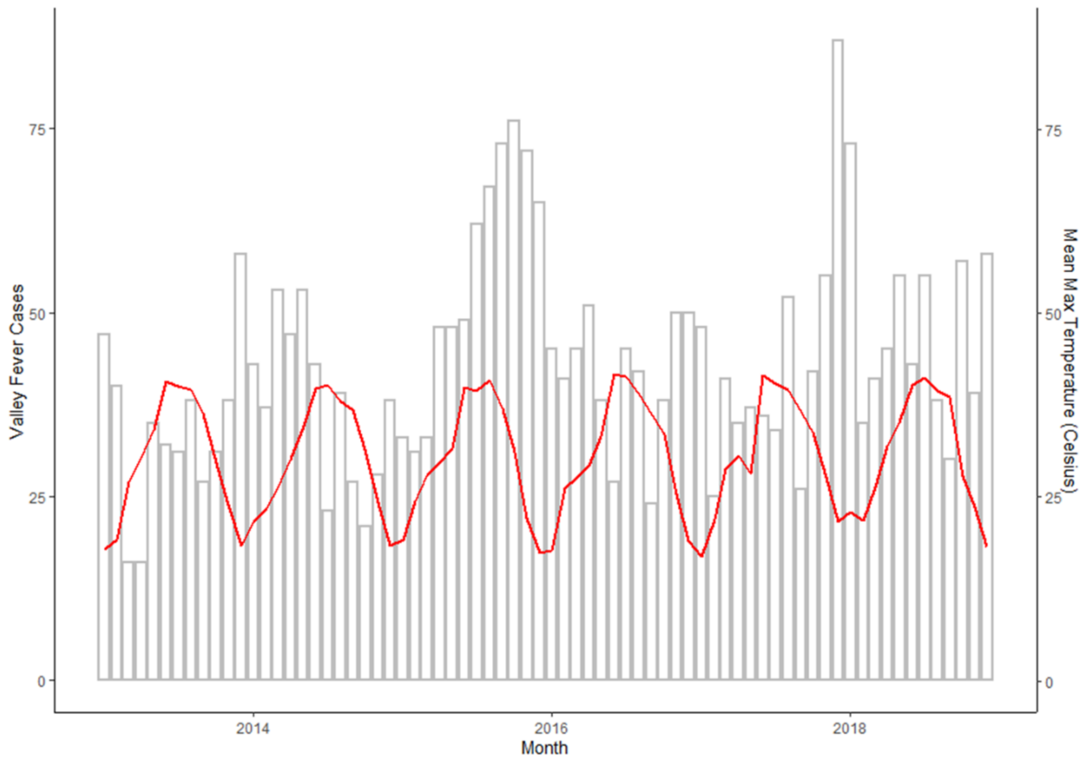
Valley Fever Cases
0
25
50
75
Mean Max Temperature (Celsius)
0
25
50
75
2014
2016
2018
Month

S1i.

Figure S1: Monthly Coccidioidomycosis reported cases 2013-2018 from Pima, Maricopa, and Pinal counties in the highly endemic region of Southern Arizona. Monthly climatic variables are plotted with the reported cases a) red line represents the monthly average of particulate matter 10 microns and smaller (PM10 ug/m^3) in Pima County, Arizona b) total monthly precipitation (mm) in Pima County, Arizona c) monthly mean maximum temperature (^{o} Celsius) in Pima County, Arizona d) monthly average PM10 (ug/m^3) in Maricopa County, Arizona e) total monthly precipitation (mm) in Maricopa County, Arizona f) monthly mean maximum temperature (^{o} Celsius) in Maricopa County, Arizona g) monthly average PM10 (ug/m^3) in Pinal County, Arizona h) total monthly precipitation (mm) in Pinal County, Arizona i) monthly mean maximum temperature (^{o} Celsius) in Pinal County, Arizona.

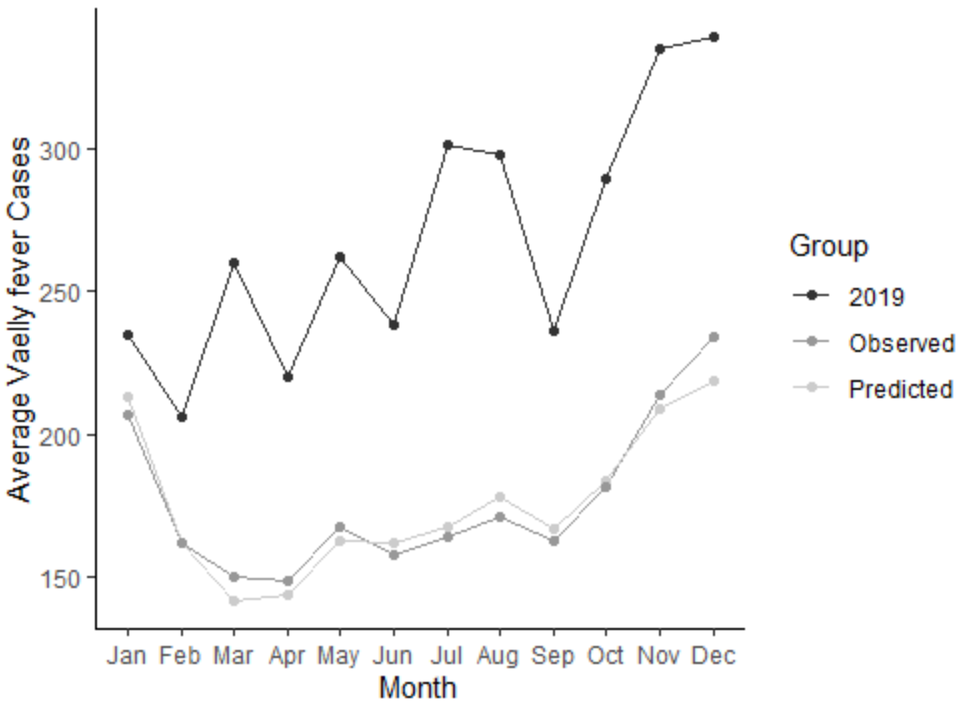

Figure S2: Average monthly Valley Fever Cases in Maricopa, Pinal, and Pima counties, Arizona. Predicted data represents our model predictions, observed represents actually Valley fever cases 2013-2018, 2019 represents new cases from 2019 not included in this study. This shows our model is predicting the seasonal patterns of Valley Fever very well.

Supplemental Data File 1. R code for GAM model

```
library(mgcv)
library(MASS)
library(gamm4)
library(tidyr)
library(ggplot2)
library(ggthemes)
library(viridis)
library(cowplot)
library(kableExtra)
library(docxtools)
library(knitr)
library(tibble)
library(dplyr)
library(gratia)
library(latex2exp)
library(ggstance)
#making year and month factors
southaz <-
read.csv("C:/Users/DKollath/OneDrive/Desktop/Rstuff/
threecounties_month.csv") %>%
mutate(year=factor(year))%>%
mutate(month=factor(month)) %>%
mutate(season=factor(season))
str(southaz)
#center and scale your covariates:
southaz =
southaz %>%
mutate(AWND_s = scale(AWND)[,1],
PRCP_s = scale(PRCP)[,1],
TAVG_s = scale(TAVG)[,1],
TMAX_s = scale(TMAX)[,1],
TMIN_s = scale(TMIN)[,1],
PM10_s = scale(PM10)[,1],
RH_s = scale(RH)[,1])
#Adding lags to PM10 and Precip
southaz=
southaz %>%
mutate(PRCP_LAG=lag(PRCP_s,2),
PM10_LAG=lag(PM10_s,2),
PM10_LLag=lag(PM10,2),
rainlag=lag(PRCP,2))
# Look for correlations:
quartz()
pairs(southaz[,c(12:18)])
# Notes:
## Tmin,Tmax,Tavg highly correlated. Must choose 1
## RH somewhat correlated with the Temps and PRCP, so probably won't
use
#model with cubic regression splines by month
```

```
#simplify to figure out what this code is actually doing
testmod2_crs<-gam(cases~
s(PRCP_s,bs="cr",by=month, k=3)+
# s(PRCP,bs="cr",by=month,k=3)+
# s(TAVG,bs="cr",by=month,k=3)+
# s(TMIN,bs="cr",by=month,k=3)+
# s(TMAX,bs="cr",by=month,k=3)+
# s(PM10,bs="cr",by=month,k=3)+
# s(AWND,bs="cr",by=month,k=3)+
# s(RH,bs="cr",by=month,k=3)+
s(county,bs="re")+
s(year,bs="re"),
data = southaz,family = nb,method = "REML")
summary(testmod2_crs)
quartz()
gratia::draw(testmod2_crs)
##################################################
##################################################
simplemod<-gam(cases~AWND_s+PRCP_s+TMAX_s+PM10_s,data = southaz)
summary(simplemod)
AIC(simplemod)
sqrt(mean(residuals.gam(simplemod,type="response")^2))
southaz$month = as.numeric(southaz$month)
testmod2_crs2<-gam(cases~
te(month,PRCP_s,
bs=c("cc","cr"), k=c(5,5))+
s(county,bs="re")+
s(year,bs="re"),
data = southaz,family = nb,method = "REML",
knots = list(month=c(1,12))) #expect Dec,Jan to be
similar cuz winter
summary(testmod2_crs2)
AIC(testmod2_crs2)
sqrt(mean(residuals.gam(testmod2_crs2,type="response")^2))
quartz()
gratia::draw(testmod2_crs2)
# Note that the grayed out region has no data in your df
# Here you can see that it looks like low PRCP is associated with more
cases, but only in winter.
other_df =
filter(southaz, month >1 & month<7)
winter_df =
filter(southaz, month <2 | month>10)
par(mfrow=c(1,2))
plot(winter_df$cases ~ winter_df$PRCP_s,
main = "Winter", xlab = "PRCP, scaled", ylab = "Cases")
plot(other_df$cases ~ other_df$PRCP_s,
main = "Other", xlab = "PRCP, scaled", ylab = "Cases")
# ADD temperature
testmod2_crs3<-gam(cases~
te(month,PRCP_s,
```

```
bs=c("cc","cr"), k=c(5,5))+
te(month,TMAX_s,
bs=c("cc","cr"), k=c(5,5))+
s(county,bs="re")+
s(year,bs="re"),
data = southaz,family = nb,method = "REML",
knots = list(month=c(1,12)))
summary(testmod2_crs3)
AIC(testmod2_crs3)
sqrt(mean(residuals.gam(testmod2_crs3,type="response")^2))
quartz()
gratia::draw(testmod2_crs3)
# low temps in the winter are associated with higher cases
# Continue adding:
testmod2_crs4<-gam(cases~
te(month,PRCP_s,
bs=c("cc","cr"), k=c(5,5))+
te(month,TMAX_s,
bs=c("cc","cr"), k=c(5,5))+
te(month,AWND_s,
bs=c("cc","cr"), k=c(5,5))+
te(month,PM10_s,
bs=c("cc","cr"), k=c(5,5))+
s(county,bs="re")+
s(year,bs="re"),
data = southaz,family = nb,method = "REML",
knots = list(month=c(1,12)))
summary(testmod2_crs4)
AIC(testmod2_crs4)
sqrt(mean(residuals.gam(testmod2_crs4,type="response")^2))
quartz(height = 7, width = 14)
gratia::draw(testmod2_crs4)
AIC(testmod2_crs4)
# Look at PM10
# Seems like High PM10 = more cases in winter, but
# High PM10 = less cases in other months
par(mfrow=c(1,2))
plot(winter_df$cases ~ winter_df$PM10_s,
main = "Winter", xlab = "PRCP, scaled", ylab = "Cases")
plot(other_df$cases ~ other_df$PM10_s,
main = "Other", xlab = "PRCP, scaled", ylab = "Cases")
###adding lags to pm10 and precip
testmod2_crs5<-gam(cases~
te(month,PRCP_s,
bs=c("cc","cr"), k=c(5,5))+
te(month,TMAX_s,
bs=c("cc","cr"), k=c(5,5))+
te(month,AWND_s,
bs=c("cc","cr"), k=c(5,5))+
te(month,PM10_s,
bs=c("cc","cr"), k=c(5,5))+
```

```
te(month,PRCP_LAG,
bs=c("cc","cr"),k=c(5,5))+
te(month,PM10_LAG,
bs=c("cc","cr"),k=c(5,5))+
s(county,bs="re")+
s(year,bs="re"),
data = southaz,family = nb,method = "REML",
knots = list(month=c(1,12)))
summary(testmod2_crs5)
summary.gam(testmod2_crs5)
AIC(testmod2_crs5)
sqrt(mean(residuals.gam(testmod2_crs5,type="response")^2))
gratia::draw(testmod2_crs5)
AIC(testmod2_crs5)
gam.check(testmod2_crs5)
concurvity(testmod2_crs5)
coef(testmod2_crs5)
AIC(testmod2_crs5,testmod2_crs4,testmod2_crs3,testmod2_crs2,testmod2_c
rs,testmod_final)
testmod2_crs6<-gam(cases~
te(month,PRCP_s,
bs=c("cc","cr"), k=c(5,5))+
te(month,TMAX_s,
bs=c("cc","cr"), k=c(5,5))+
te(month,AWND_s,
bs=c("cc","cr"), k=c(5,5))+
te(month,PM10_s,
bs=c("cc","cr"), k=c(5,5))+
s(county,bs="re")+
s(year,bs="re")+
te(month,PRCP_LAG, bs=c("cc","cr"),k=c(5,5)),
data = southaz,family = nb,method = "REML", select
= T,
knots = list(month=c(1,12)))
summary(testmod2_crs6)
AIC(testmod2_crs5,testmod2_crs6)
##################################################
##################################################
# simulation:
testmod_final <-gam(cases~
te(month,PM10_s,
bs=c("cc","cr"), k=c(5,5))+
te(month,PM10_LAG, bs=c("cc","cr"),k=c(5,5))+
te(month,PRCP_LAG, bs=c("cc","cr"),k=c(5,5))+
te(month,TMAX_s,
bs=c("cc","cr"), k=c(5,5))+
s(county,bs="re")+
s(year,bs="re"),
data = southaz,family = nb,method = "REML",
knots = list(month=c(1,12)))
summary(testmod_final)
```

```
quartz()
gratia::draw(testmod_final)
AIC(testmod_final,testmod2_crs5)
# Simulate new data (the full range of PM10_s)
# Imagine you have observations across the range of PM10 for every
month, every year, and every county
n_obs = 100
sim_PM10_s = seq(min(southaz$PM10_s), max(southaz$PM10_s), length.out
= n_obs)
n_county = length(unique(southaz$county))
sim_month = seq(1, 12, length.out = 30)
n_month = length(sim_month)
n_year = length(unique(southaz$year))
## simulate across full range of PM10_lag,Precip_lag, and TAVG
southaz<-na.omit(southaz)
sim_PM10_LAG = seq(min(southaz$PM10_LAG), max(southaz$PM10_LAG),
length.out = n_obs)
sim_PRCP_LAG = seq(min(southaz$PRCP_LAG), max(southaz$PRCP_LAG),
length.out = n_obs)
sim_TMAX_s = seq(min(southaz$TMAX_s), max(southaz$TMAX_s), length.out
= n_obs)
sim_PRCP_s=seq(min(southaz$PRCP_s), max(southaz$PRCP_s), length.out =
n_obs)
sim_AWND_s=seq(min(southaz$AWND_s), max(southaz$AWND_s), length.out =
n_obs)
# Now needed a nested data set:
new_df =
data.frame(
year = rep(unique(southaz$year),
each = n_county*n_month*n_obs),
county= rep(
rep(unique(southaz$county), each = n_month*n_obs),
times = n_year),
month = rep(
rep(sim_month, each = n_obs),
times = n_year*n_county),
PM10_s = rep(sim_PM10_s, times = n_year*n_county*n_month),
PM10_LAG= rep(sim_PM10_LAG,times = n_year*n_county*n_month),
PRCP_LAG= rep(sim_PRCP_LAG, times= n_year*n_county*n_month),
TMAX_s= rep(sim_TMAX_s,times= n_year*n_county*n_month),
PRCP_s=rep(sim_PRCP_s,times=n_year*n_county*n_month),
AWND_s=rep(sim_AWND_s,times=n_year*n_county*n_month)
)
test_pred = predict(testmod2_crs5, newdata = new_df,
type = "terms") ##try with the best model
str(test_pred)
#Add the effect of te(month,PM10_s)
new_df$mod_pred = test_pred[,2]
new_df$mod_pred = test_pred[,4]
new_df$mod_pred = test_pred[,5]
new_df$mod_pred = test_pred[,6]
```

```
# Generate a simliar plot as gratia::draw()PM10_s plot
quartz(height = 4, width = 4.5)
ggplot(new_df) +
geom_tile(aes(x = month, y = PM10_s, fill = mod_pred)) +
geom_contour(aes(x = month, y = PM10_s, z = mod_pred),
color = "black") +
scale_fill_gradient2(name = "Effect",
low = "dodgerblue3", mid="white",high = "red3",
midpoint = 0.00,limits=c(-1.5,1.5)) +
theme_classic()+xlab("Month")+ylab("Scaled PM10")
+scale_x_discrete(limits=month.abb)
# PM10_LAG Plot
ggplot(new_df) +
geom_tile(aes(x = month, y = PM10_LAG, fill = mod_pred)) +
geom_contour(aes(x = month, y = PM10_LAG,z = mod_pred),
color = "black") +
scale_fill_gradient2(name = "Effect",
low = "dodgerblue3", mid="white",high = "red3",
midpoint = 0.00,limits=c(-1.5,1.5)) +
theme_classic()+xlab("Month")+ylab("Scaled Lagged PM10")
+scale_x_discrete(limits=month.abb)
#PRCP_lag Plot
ggplot(new_df) +
geom_tile(aes(x = month, y = PRCP_LAG, fill = mod_pred)) +
geom_contour(aes(x = month, y = PRCP_LAG,z = mod_pred),
color = "black") +
scale_fill_gradient2(name = "Effect",
low = "dodgerblue3", mid="white",high = "red3",
midpoint = 0.0,limits=c(-1.5,1.5)) +
theme_classic()+xlab("Month")+ylab("Scaled Lagged Total
Precipitation")+scale_x_discrete(limits=month.abb)
#TMAX Plot
ggplot(new_df) +
geom_tile(aes(x = month, y = TMAX_s, fill = mod_pred)) +
geom_contour(aes(x = month, y = TMAX_s,z = mod_pred),
color = "black") +
scale_fill_gradient2(name = "Effect",
low = "dodgerblue3", mid="white",high = "red3",
midpoint = 0.0,limits=c(-1.5,1.5)) +
theme_classic()+ xlab("Month")+ ylab("Scaled Mean Max Temperature")
+scale_x_discrete(limits=month.abb)
##plotting raw data to see relationships
view(southaz)
ggplot(southaz, aes(x = PM10, y = cases, color = season)) +
geom_point() +
labs(x = "PM10", y = "Valley fever Cases",
color = "Seasons\nindicated\nby colors:")
month.labs<-
c("Jan","Feb","March","April","May","June","July","Aug","Sep","Oct","N
ov","Dec")
names(month.labs)<-
```

```
c("1","2","3","4","5","6","7","8","9","10","11","12")
ggplot(data=southaz, aes(x=PM10, y=cases,group=month)) +
facet_wrap(~month,scales="free_x",labeller =
labeller(month=month.labs)) +
geom_point() +labs(x = "PM10", y = "Valley fever Cases",
color = "Seasons\nindicated\nby colors:") +
geom_smooth(method='loess',span=2, formula= y~x)+theme_bw()
?geom_smooth
##all together
ggplot(data=southaz, aes(x=PM10, y=cases)) +
geom_point() +labs(x = "PM10", y = "Valley fever Cases",
color = "Seasons\nindicated\nby colors:") +
geom_smooth(method='loess', formula= y~x)+theme_bw()
ggplot(data=southaz, aes(x = TMAX, y = cases, color = season)) +
geom_point() +
labs(x = "Mean Max Temperature (C)", y = "Valley fever Cases",
color = "Seasons\nindicated\nby colors:")
ggplot(data=southaz, aes(x=TMAX, y=cases,group=month)) +
facet_wrap(~month,scales="free_x",labeller =
labeller(month=month.labs)) +coord_cartesian(ylim = c(0,750))+
geom_point() +labs(x = "Mean Max Temperature (C)", y = "Valley fever
Cases",
color = "Seasons\nindicated\nby colors:")
+theme_bw()+
geom_smooth(method='loess',span=3, formula= y~x)
##all together
ggplot(data=southaz, aes(x=TMAX, y=cases)) +
geom_point() +labs(x = "Mean Max Temperature (C)", y = "Valley fever
Cases",
color = "Seasons\nindicated\nby colors:")
+theme_bw()+
geom_smooth(method='loess', formula= y~x)+ylim(0,800)
ggplot(data=southaz, aes(x =PM10_LLag , y = cases, color = season)) +
geom_point() +
labs(x = "Lagged PM10", y = "Valley fever Cases",
color = "Seasons\nindicated\nby colors:")
ggplot(data=southaz, aes(x=PM10_LLag, y=cases,group=month)) +
facet_wrap(~month,scales="free_x",labeller =
labeller(month=month.labs)) +
geom_point() +labs(x = "Lagged PM10", y = "Valley fever Cases",
color = "Seasons\nindicated\nby colors:")
+theme_bw()+
geom_smooth(method='loess',span=2, formula= y~x)
##all together
ggplot(data=southaz, aes(x=PM10_LLag, y=cases)) +
geom_point() +labs(x = "Lagged PM10", y = "Valley fever Cases",
color = "Seasons\nindicated\nby colors:")
+theme_bw()+
geom_smooth(method='loess', formula= y~x)
ggplot(data=southaz, aes(x =rainlag , y = cases, color = season)) +
geom_point() +
```

```
labs(x = "Lagged Precipitaion (mm)", y = "Valley fever Cases",
color = "Seasons\nindicated\nby colors:")
ggplot(data=southaz, aes(x=rainlag, y=cases,group=month)) +
facet_wrap(~month,scales="free_x",labeller =
labeller(month=month.labs)) +
geom_point() +labs(x = "Lagged Precipitation (mm)", y = "Valley
fever Cases",
color = "Seasons\nindicated\nby colors:")
+theme_bw()+
geom_smooth(method='loess',span=2, formula= y~x)+ylim(0,1000)
##all together
ggplot(data=southaz, aes(x=rainlag, y=cases)) +
geom_point() +labs(x = "Lagged Precipitation (mm)", y = "Valley
fever Cases",
color = "Seasons\nindicated\nby colors:")
+theme_bw()+
geom_smooth(method='loess', formula= y~x)+ylim(0,1000)
```
